# Dr. Swan and the I. H. A.

**Published:** 1887-05

**Authors:** 


### DR. SWAN AND THE I. H. A.

"Personal interests or ambitions have no place, but only *what* is truth."

The International Hahnemannian Association was organized for the study and perfection of Homœopathy by such physicians as believed in Hahnemann's teachings. In the performance of this work the Association has been compelled to fight error upon all sides. It has not shrunken in the past from boldly combating all false teachings which it has found. Shall it now avoid the performance of a duty to the science it loves, because such duties necessitate the repudiation of false teaching by one of its own members? The duty is unpleasant, but we hope the I. H. A. has the courage to perform it.

Dr. S. Swan has for many years held and openly advocated medical (if such they can be called?) opinions which are at variance with Hahnemannian Homœopathy. He has been tolerated until forbearance ceases to be a virtue. In his latest publication his absurdities have passed all bounds. We refer to the *Catalogue of Morbific Products, Nosodes, and other Remedies,* which he has published and dared, with unwarranted impudence, to stamp with the seal of the I. H. A. It would thereby seem as if the Association authorized the publication of this *Catalogue.*

It is scarcely necessary to quote any evidence of its quackery; but we may be excused for asking if his "quadruple mixture," or his mixture of twelve tissue remedies, be homœopathic, upon what symptoms can they be prescribed? Or, can one imagine how he could use the "Seriaca Barlowii," which Dr. Swan kindly informs the public is "from a silk handkerchief eaten by a cow, and taken from the stomach in a hard ball."

Moreover, at page thirty-one, "a generalization" is given which is *untrue.* It says: "Morbific matter will cure the disease which produced it, if given in a high potency, even to the person from whom it was obtained."

Neither Dr. Swan nor any one else has cured enough of such cases to prove anything. We have heard of many cases Dr. S. has treated which have not been benefited; rather the reverse. But it is useless to discuss the question.

Dr. Swan will continue to advocate this empiricism, for he believes himself to be a great discoverer, and to be persecuted as many really great men have been persecuted for their discoveries. Dr. Swan has not discovered *anything,* but has taken up the ideas of others, and has gone wild over them, a veritable Don Quixote!

With the opinions and the acts of Dr. Swan as an individual we have naught to do, but when he or any one else publishes a catalogue of such nonsense under the seal of the I. H. A., then it behooves us to speak out plainly. We assert that Dr. Swan must leave the I. H. A., and his *opinions must be condemned*, or the I. H. A. will become a caricature. Dr. Hering wrote long ago: "If our school ever gives up the strict inductive method of Hahnemann, we are lost, and deserve only to be mentioned as a caricature in the history of medicine." Dr. Swan's methods are at variance in every way with "Hahnemann's strict inductive method." Which shall it be—Hahnemann or Swan, Homœopathy or empiricism? The I. H. A. cannot advocate both, they are as opposite as day and night, as truth and falsehood.

